# Protocol for Correcting Residual Errors with Spectral, ULtrasound, Traditional Speech therapy Randomized Controlled Trial (C-RESULTS RCT)

**DOI:** 10.1186/s12887-020-1941-5

**Published:** 2020-02-11

**Authors:** Tara McAllister, Jonathan L. Preston, Elaine R. Hitchcock, Jennifer Hill

**Affiliations:** 1 Department of Communicative Sciences and Disorders, New York University, New York, NY USA; 2 Department of Communication Sciences and Disorders, Syracuse University, 621 Skytop Rd, Suite 1200, Syracuse, NY 13244 USA; 3 Department of Communication Sciences & Disorders, Montclair State University, Bloomfield, NJ USA; 4 Department of Applied Statistics, Social Science, and the Humanities, New York University, New York, NY USA

**Keywords:** Speech sound disorder, Randomized controlled trial, Biofeedback

## Abstract

**Background:**

Speech sound disorder in childhood poses a barrier to academic and social participation, with potentially lifelong consequences for educational and occupational outcomes. While most speech errors resolve by the late school-age years, between 2 and 5% of speakers exhibit residual speech errors (RSE) that persist through adolescence or even adulthood. Previous findings from small-scale studies suggest that interventions incorporating visual biofeedback can outperform traditional motor-based treatment approaches for children with RSE, but this question has not been investigated in a well-powered randomized controlled trial.

**Methods/design:**

This project, *Correcting Residual Errors with Spectral, ULtrasound, Traditional Speech therapy Randomized Controlled Trial* (*C-RESULTS RCT*), aims to treat 110 children in a parallel randomized controlled clinical trial comparing biofeedback and non-biofeedback interventions for RSE affecting the North American English rhotic sound /ɹ/. Eligible children will be American English speakers, aged 9–15 years, who exhibit RSE affecting /ɹ/ but otherwise show typical cognitive-linguistic and hearing abilities. Participants will be randomized, with stratification by site (Syracuse University or Montclair State University) and pre-treatment speech production ability, to receive either a motor-based treatment consistent with current best practices in speech therapy (40% of participants) or treatment incorporating visual biofeedback (60% of participants). Within the biofeedback condition, participants will be assigned in equal numbers to receive biofeedback in the form of a real-time visual display of the acoustic signal of speech or ultrasound imaging of the tongue during speech. The primary outcome measure will assess changes in the acoustics of children’s production of /ɹ/ during treatment, while a secondary outcome measure will use blinded listeners to evaluate changes in the perceived accuracy of /ɹ/ production after the completion of all treatment. These measures will allow the treatment conditions to be compared with respect to both efficacy and efficiency.

**Discussion:**

By conducting the first well-powered randomized controlled trial comparing treatment with and without biofeedback, this study aims to provide high-quality evidence to guide treatment decisions for children with RSE.

**Trial registration:**

ClinicalTrials.gov identifier NCT03737318, November 9, 2018.

## Background

Developmental speech sound disorder results in reduced speech intelligibility and poses a barrier to academic and social participation. The negative socioemotional ramifications of speech sound disorder have been well documented [1, 2], and the impact on educational and occupational outcomes may be lifelong [3, 4]. Most children with delayed speech go on to develop normal speech by 8–9 years old, but between 2 and 5% of speakers exhibit residual speech errors (RSE) that persist through adolescence or even adulthood [5–7]. RSE is known to be particularly challenging to treat, with the result that speech-language pathologists (SLPs) frequently discharge these clients with their errors uncorrected [8]. In North American English, residual errors affecting the rhotic sound /ɹ/ (as in the beginning of the word *red*) are considered the most common form of RSE [8]; in order to select a relatively homogeneous participant population, the present study focuses on this subset of individuals with RSE.

Recent evidence suggests that visual biofeedback, which uses instrumentation to provide real-time information about aspects of speech that are typically outside the speaker’s conscious control [9], can be used to enhance intervention for RSE and other speech disorders. Visual biofeedback can incorporate various technologies. The focus in C-RESULTS RCT is on two specific technologies: *ultrasound biofeedback*, in which an ultrasound probe held beneath the chin is used to create a dynamic image of the shape and movements of the tongue, and *visual-acoustic biofeedback*, in which a microphone and software are used to generate a real-time display of the resonant frequencies of the vocal tract (formants). In either type of biofeedback, the learner is shown a model or template representing correct production of the target sound and is encouraged to adjust their own output to achieve a better match with the target in the real-time visual display.

A number of recent studies have documented positive responses to biofeedback treatment in individuals who showed minimal response to previous intervention. This is true of both visual-acoustic [10–12] and ultrasound [13–16] types of biofeedback. Many of these studies have used single-case experimental methods to compare gains in biofeedback-enhanced treatment versus traditional forms of intervention. In the context of visual-acoustic biofeedback, a study of 11 children who received an initial period of traditional motor-based treatment followed by biofeedback treatment found that significant improvements in perceptual and acoustic measures of /ɹ/ production occurred only after biofeedback treatment had begun for all but one participant. Another single-case experimental study of 11 children [11] found a significant interaction between treatment condition and order, such that a period of visual-acoustic biofeedback followed by a period of traditional motor-based treatment was associated with significantly larger effect sizes than the same treatments provided in the reverse order. Finally, in a single-case randomization study [12], three out of seven participants showed a statistically significant advantage for visual-acoustic biofeedback over traditional treatment, while none showed a significant advantage in the opposite direction.

In a recent systematic review of 28 ultrasound biofeedback treatment studies reporting on over 100 participants, Sugden et al. [17] indicated that this approach resulted in positive outcomes in many, but not all, children. For example, a single-case study with 8 children [16] found that participants who previously had not responded to as much as 11 years of traditional intervention achieved large average effect sizes in ultrasound biofeedback treatment. Another recent study [15] reported that improvements in /ɹ/ production were significantly greater for six children who completed 8 sessions with ultrasound biofeedback therapy than for six children who had completed 8 sessions of traditional articulation treatment.

The main limitation of previous research is the small number of individuals tested in each study and the lack of studies using a randomized controlled trial (RCT) methodology. Specifically, to determine whether biofeedback is an operative component of treatment, it is important to compare similarly structured treatment programs that differ only in the presence of biofeedback. In this study, therefore, biofeedback is compared to a similarly structured non-biofeedback treatment, termed motor-based treatment (MBT). Another limitation of previous research pertains to the outcome measures used to evaluate treatment response. While most published studies have focused on evaluating *generalization* of treatment gains (e.g., improvement in untreated words or contexts), models of motor learning suggest that biofeedback, as a form of detailed qualitative feedback, should have its greatest impact in early phases of treatment (*acquisition*) [18, 19]. For example, in one study [14], participants produced correct trials at a rate of 229 trials per hour in an ultrasound treatment condition, which significantly exceeded the rate of 155 correct trials per hour in the non-biofeedback treatment condition. However, by the end of the study, both conditions yielded roughly similar levels of generalization. Thus, studies comparing biofeedback and non-biofeedback treatment should consider the possibility that the largest difference may lie in the area of *efficiency* rather than *efficacy*.

C-RESULTS RCT aims to address these limitations by conducting a well-powered RCT comparing biofeedback and non-biofeedback forms of intervention. Visual-acoustic and ultrasound biofeedback, which have comparable evidence of efficacy, will be represented equally in the biofeedback condition. The non-biofeedback treatment condition, MBT, also has documented evidence of efficacy [20, 21]. With treatment protocols and outcome measures that have been refined over numerous small experimental studies, this RCT can be expected to provide interpretable evidence for or against our hypothesis of improved outcomes in biofeedback relative to non-biofeedback intervention. To assess the possibility of differences in either efficiency or efficacy, the present study will track two outcomes. Acquisition, or speech performance over the course of early treatment, will serve as the primary outcome measure. Generalization, or speech performance on untrained contexts without clinician support, will serve as a secondary measure. Based on previous findings, we hypothesize that both MBT and biofeedback groups will improve over the course of treatment, but we also predict a significant between-group difference whereby pre-post gains associated with biofeedback treatment will significantly exceed those observed in the non-biofeedback MBT condition. We hypothesize that these differences will affect measures of both acquisition and generalization; if we observe a difference in the former but not the latter, this will constitute evidence that biofeedback is more efficient, but not more effective, than non-biofeedback treatment.

## Methods and design

C-RESULTS RCT is a parallel-group randomized controlled trial measuring the efficiency and efficacy of intervention with and without biofeedback for children with RSE affecting /ɹ/. Participants will be assigned to receive a standard dose of intervention in a biofeedback treatment condition or in the MBT (non-biofeedback) condition. Individuals assigned to the biofeedback treatment condition will be sub-allocated to receive either visual-acoustic or ultrasound biofeedback treatment in equal numbers. A comparison of biofeedback types is proposed as part of a separate study but will not constitute a primary aim of C-RESULTS RCT. To maximize power for the separate study comparing biofeedback types, participants will be allocated to biofeedback versus MBT conditions in a 3:2 ratio. Participant allocation in each group will be further stratified by pre-treatment severity, since previous research has identified this variable as an important indicator of subsequent treatment response. Finally, allocation will additionally be stratified by site, as data collection will occur in two locations in the United States (Montclair, New Jersey; Syracuse, New York).

We plan to enroll a total of 110 children with RSE. Our previous work comparing biofeedback and non-biofeedback treatment found a median effect size (Cohen’s d) of .70 for MBT versus ultrasound treatment [14] and .64 for MBT versus visual-acoustic biofeedback [11]. Because both biofeedback types will be represented in the biofeedback treatment condition in C-RESULTS RCT, we use .67 as our best estimate of the likely effect size of the difference between biofeedback and MBT conditions. Calculations in G*Power [22] indicate that our proposed sample of 110 children has > 90% power to detect an effect of this magnitude. This power calculation was based on a fully randomized experimental design; however, our final design includes blocking on pre-treatment accuracy, which we expect to be a significant predictor of outcomes. Thus, the original power calculation can be assumed to represent a conservative estimate.

### Participants

Ethics approval for this study has been obtained through the Biomedical Research Association of New York (BRANY, protocol #18–10-393). C-RESULTS RCT is a multi-site study with two treatment sites (Montclair State University and Syracuse University) and a central site for data processing and analysis (New York University). All participants will provide written assent to take part in the study, and the parent/guardian will additionally provide written permission for participation. For secure management of potentially identifying information, including consent/assent and responses to questionnaires, all study data will be entered and verified in REDCap electronic data capture tools hosted at Syracuse University [23, 24]. Participants will be recruited primarily through community speech-language pathologists (SLPs). Cooperating SLPs will be identified mainly through online postings to listservs, blogs, social media channels, alumni lists, and personal contacts. Other participants may be referred directly by their parents, who will be reached by announcements posted to parenting groups on listservs and social media, as well as flyers posted in public locations such as libraries, schools, and pediatricians’ offices. Participant enrollment began August 29, 2019.

All participants must speak English as a dominant language and must have begun learning English by age 3, per parent report on a questionnaire. They must have no history of sensorineural hearing loss, major neurobehavioral disorder, or developmental disability (e.g., autism spectrum disorder, Down Syndrome), as indicated by parent report. They must be between 9;0 and 15;11 years of age at the time of enrollment. Participants must also pass a pure-tone hearing screening at 20 dB HL and a brief examination of oral structure and function. To rule out nonverbal cognitive delays, participants must additionally demonstrate adequate nonverbal skills as defined by a T score < 1.3 SD below the mean on the Wechsler Abbreviated Scale of Intelligence-2 (WASI-2) [25] Matrix Reasoning. To rule out cases of language impairment, which could confound the interpretation of our results, all participants must have language skills broadly within normal limits as evidenced by a passing score on the Clinical Evaluation of Language Fundamentals-5 (CELF-5) [26] screening measure, or a minimum standard score of 80 on the Core Language Index of the CELF-5 (see below). Finally, to select individuals with a relatively uniform level of baseline severity, participants must exhibit less than 30% accuracy, based on consensus across 2 trained listeners, on a 50-item probe list eliciting /ɹ/ in various words. They must also score no higher than the 8th percentile on the Goldman-Fristoe Test of Articulation-3 (GFTA-3) [27].

To rule out childhood apraxia of speech (CAS), we will initially administer two tasks; participants who score in the range for childhood apraxia on both tasks will be automatically excluded. (1) The Syllable Repetition task will be used to determine the number of added segments, with a cutoff score of 4 or more additions reflecting likely CAS [28]. (2) The multisyllabic word task of the LinguiSystems Articulation Test [29] will be administered to identify the number of inconsistencies across repeated productions, with a cutoff score of three or more inconsistencies reflecting likely CAS. If participants pass one measure but fail the other, a maximum performance task will be administered as a tiebreaker measure. In the maximum performance task, children sustain phonemes (e.g., /a/, /s/), repeat single syllables (e.g., /papapa/), and repeat trisyllable sequences (/pataka/). Procedures outlined by Thoonen et al. [30, 31] will be followed to identify suspected cases of CAS based on slow or inaccurate trisyllable production. Participants who do not achieve a passing score on two of the three measures will be excluded.

No participants will be excluded from the proposed study on the basis of sex/gender or racial/ethnic group. Both male and female children will be recruited, and the large proposed sample size will allow us to test for any influence of gender on either typical performance patterns or response to intervention. However, based on the general makeup of the population of children with speech sound disorders [5], we anticipate that slightly more males will be referred than females.

### Assessment process

A phone screening will initially be conducted to identify any exclusionary criteria identifiable via parent report (e.g., outside of age range, not a native speaker of English, or diagnosis of developmental disability). A detailed description of study requirements will also be presented to parents at this time. Individuals who pass the phone screening will be invited to participate in a 1–2 h eligibility assessment. Consent and assent instruments will be administered at this time, as well as questionnaires collecting detailed information about participants’ health and language history. The following tasks will be administered: hearing screening, oral mechanism screening, WASI-2 Matrix Reasoning [25], CELF-5 Screener [26], GFTA-3 [27], Syllable Repetition Task [28], and Linguisystems Articulation Test Inconsistency Screener [29]. Participants will also produce custom probes eliciting /ɹ/ at the syllable/disyllable level (stimulability probe [32]) and word level. Individuals who pass all eligibility criteria will be asked to return for 1–2 additional testing sessions that will collect information about acuity in auditory and somatosensory domains, to be used as part of a separate analysis examining individual predictors of response to treatment. If the results of any preliminary testing were equivocal, additional eligibility testing will be carried out at this point. Specifically, the full CELF-5 Core Language Index will be administered if the CELF-5 screening measure was not passed, and the above-described maximum performance tasks will be administered if the participant passed one but not both of the standard screening measures for CAS.

### Group allocation

Following the initial evaluation visits, all participants will complete a Dynamic Assessment session (Phase 0) consisting of 2 h of non-biofeedback intervention, described in more detail below, in which the clinician will provide extensive cueing and feedback in an effort to elicit the client’s best attempts at /ɹ/ production. Based on the treating clinicians’ perceptual ratings of participants’ performance in Phase 0, participants will be categorized as high responders (> 5% accuracy during Dynamic Assessment) or low responders (0–5% accuracy during Dynamic Assessment). The study statistician will generate confidential participant treatment assignments in batches of 10, where each batch of 10 corresponds to a combination of site (Montclair State University versus Syracuse University) and response category (high versus low). Random assignments will be generated to ensure the following distribution per batch: 3 individuals assigned to receive visual-acoustic biofeedback, 3 to receive ultrasound biofeedback, and 4 to receive MBT. The first 4 batches will correspond to the first 10 participants recruited in each of these site-by-category combinations. The second 4 batches will correspond to the same site-by-category combinations for the next time period. The PIs will monitor allocation of participants to response categories within each site to understand whether the distribution is reasonably balanced. If it is, the final 4 batches will be assigned similarly. If not, the treatment assignment may be altered.

In cases where a participant drops out prior to the completion of Phase 1 (see below), the next participant to enroll will be assigned to the same treatment condition as a replacement for the dropout. If a participant drops out after the completion of Phase 1, they will retain their random assignment and the next participant will receive a new assignment. Dropouts will be offered compensation to return for a follow-up assessment after the typical duration of treatment elapses, in order to measure their outcomes in the absence of treatment.

### Intervention delivery and dosage

All treatment will be provided in an individualized fashion by a certified SLP who will have completed a standard training process, detailed below. Treatment will take place in research space at Syracuse University or Montclair State University. While participating in the study intervention, participants who are enrolled in any outside speech therapy will be asked to discontinue treatment on /ɹ/ if possible.

**Fig. 1:**
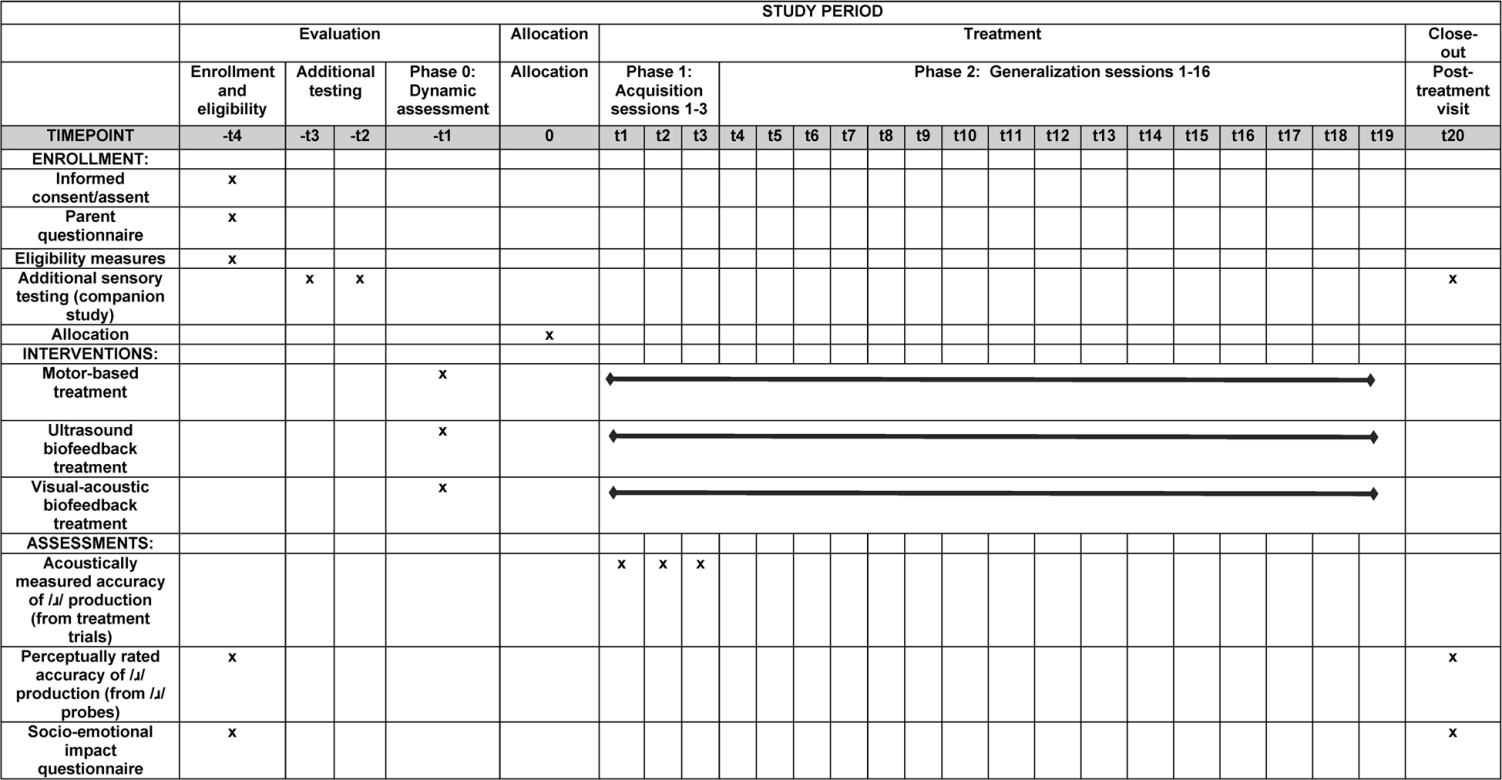
Schedule of evaluation, allocation, treatment, and close-out activities for C-RESULTS RCT

The data collection schedule is outlined in Fig. 1. Independent of a participant’s assigned treatment condition, interventions will be divided into three phases, each with its own schedule and dosage of treatment. Prior to randomization, all participants will complete a single 90-min Dynamic Assessment session featuring non-biofeedback treatment (Phase 0). The goal of this session is to evaluate participants’ stimulability for /ɹ/ production and classify them into the high- and low-response categories used for stratification in randomization, as described above. The Dynamic Assessment session will feature approximately 25 min of detailed instruction on the articulatory characteristics of English /ɹ/, followed by 15–20 min of unstructured pre-practice aimed at eliciting /ɹ/ in various syllables using a standard set of non-biofeedback techniques (verbal models, phonetic placement cues, and shaping strategies). This will be followed by a period of structured practice, approximately 45 min in duration, eliciting up to 200 syllables. The session will be terminated after 200 syllables are produced or after the cumulative session duration reaches 90 min.

After randomization, participants will complete two additional phases of treatment in their allocated condition. Phase 1 (Acquisition) will consist of 1 week of high-intensity, highly interactive practice, while Phase 2 (Generalization) will involve a longer period of lower-intensity, more structured practice. Specifically, Phase 1 (Acquisition) will focus on eliciting /ɹ/ at the syllable level in three 90-min sessions delivered over the course of 1 week. The first 45 min of these three sessions will consist of relatively unstructured pre-practice, similar to that described above for Phase 0 (Dynamic Assessment), but with the addition of condition-specific prompts and feedback. In the first Acquisition session only, participants will receive detailed condition-specific instructions, described in detail below, for approximately 25 of the first 45 min. The second half of the session will consist of structured practice; as described above for Phase 0 (Dynamic Assessment), structured practice will terminate after 200 syllables are elicited or after cumulative session duration reaches 90 min.

Phase 2 (Generalization) will consist of 16 semiweekly sessions with a duration of 45 min to 1 h. Each session in Phase 2 will begin with up to 10 min of unstructured pre-practice similar to that provided during Phase 1. This will be followed by a period of structured practice that will terminate after 250 trials or after cumulative session duration reaches 1 h, whichever comes first. In an effort to maximize generalization of learning gains, the difficulty of practice in Phase 2 will be dynamically adjusted based on participant performance; we describe the procedure for adaptive difficulty in a subsequent section.

### Types of intervention

#### Motor-based treatment (MBT)

Participants across all arms of the study will be exposed to MBT because it forms the basis of the pre-randomization Phase 0 (Dynamic Assessment) session. In this initial session, participants will receive their first introduction to articulatory anatomy and tongue shapes for /ɹ/, following a script made available through an Open Science Framework repository linked in the 'Availability of data and materials' section below; completing the script takes roughly 20–25 min. Images and diagrams will be used to teach participants to identify different components of the tongue (root, back, blade, tip), with the rationale that more precise language to talk about articulation can help make articulator placement cues more effective. They will then be familiarized, via pictures, with major characteristics of correct tongue shapes for /ɹ/. They will be told that various different tongue shapes are associated with successful /ɹ/ production, but a few characteristics are shared across tongue shapes: (a) retraction of the tongue root, (b) elevation of the tongue front (tip, blade, and/or anterior dorsum), and (c) bracing of the lateral margins of the tongue against the posterior palate, with a groove down the center of the tongue. They will be instructed to compare and contrast images with and without these articulatory components, followed by comprehension questions in which they will be asked to identify correct versus incorrect tongue shapes for /ɹ/.

After the initial instructional period,^1^ the clinician will attempt to elicit correct /ɹ/, first in an unstructured pre-practice context and then in structured practice, as stated above. In pre-practice, the basic therapeutic exchange will involve providing auditory models, eliciting imitation, and offering feedback and cues to shape production into successively closer approximations of the adult target [14, 16, 33, 34]. Shaping can involve verbal cues for articulator placement (e.g., “*Pull the back of your tongue way into the back of your throat*”; “*Pretend you’re holding a marble between the tongue and the back of your throat”*) or eliciting from facilitative contexts (e.g., pairing /ɹ/ with the low back vowel /ɑ/, which is characterized by a position of the tongue body and root that is compatible with the articulatory configuration for /ɹ/). Suggested cues are summarized in a standard list that is linked through Open Science Framework. Structured practice will focus on cueing repetitive motor practice in an effort to make improved production habitual, with verbal feedback to reinforce correct productions and reshape off-target productions.

Across phases and treatment conditions, magnetic resonance (MR) images representing correct articulation of /ɹ/ will be made available to instruct the client on correct positioning of the articulators. The image used in a given session for each participant will be selected to highlight specific aspects of tongue shape, such as elevation of the tongue blade or lowering of the tongue dorsum, judged to be appropriate for improving that individual’s /ɹ/ production. (In the MBT and visual-acoustic biofeedback conditions, while the clinician lacks direct knowledge of the client’s articulator position, inferences can be drawn from the perceptual quality of their production.) Note that static images from various technologies (MR, electropalatography) are incorporated into all treatment conditions, including MBT; it is only real-time feedback that is limited to the biofeedback conditions.

#### Ultrasound biofeedback

In the ultrasound biofeedback condition, the core elements of MBT (auditory models and verbal descriptions of articulator placement) will be supplemented with a real-time ultrasound display of the shape and movements of the tongue, which can be compared against a target representing correct production. Ultrasound biofeedback will be delivered using an EchoBlaster 128 or a MicroUS probe (Telemed Medical Systems). The ultrasound hardware will be paired with Echo Wave software operating on a personal computer.

In the first session of Phase 1 (Acquisition), participants will receive an introduction to ultrasound biofeedback, roughly 20–25 min in duration, guided by a script that is available through the project’s Open Science Framework repository. Participants will be oriented to basic information about ultrasound technology (e.g., how the probe is held) and the ultrasound image (e.g., how the tongue appears in an ultrasound image, which side is the front versus the back of the tongue). They will also be taught to connect what they see in the ultrasound image with the articulatory information that was introduced in the previous session. For instance, they will identify the different parts of their tongue (tip, blade, dorsum, root) in the ultrasound image. They will discuss the major features of articulation of /ɹ/ as seen in ultrasound and review images of correct and incorrect tongue shapes for /ɹ/. As a comprehension check, participants will be asked to distinguish between ultrasound images of correct and incorrect /ɹ/.

Pre-practice and structured practice in the ultrasound biofeedback condition will be much the same as in the non-biofeedback treatment condition; the same list of suggested cues described above for the MBT condition will be used. The main difference is that, when the clinician cues the child to do something with the tongue (e.g., retract the tongue root), the ultrasound image will provide direct evidence as to whether or not the child was successful in following that instruction. In addition, the clinician will cue the child to match a visible target. One or two target tongue shapes will be selected for each participant, and a trace of the selected target will be superimposed over the ultrasound screen. Participants will be cued to reshape the tongue to match this target during /ɹ/ production. Participants will not be locked into a single tongue shape throughout the duration of treatment: if there is a lack of progress, operationalized as failure to increase by one level of difficulty across three sessions, a contrasting tongue shape will be introduced in the next session. Conversely, if a participant achieves perceptually accurate production of /ɹ/ in the treatment setting, the standard tongue shape can be replaced with a trace of the participant’s own best approximation to date.

#### Visual-acoustic biofeedback

In visual-acoustic biofeedback, again, the core elements of MBT remain unchanged, but practice is supplemented with a real-time display of the acoustic signal of speech, which can be compared against a target representing the acoustics of a correct /ɹ/. Visual-acoustic biofeedback will be delivered using the Sona-Match module of the PENTAXMedical Computerized Speech Lab (CSL) software, which will be paired with CSL hardware model 4500B.

In the first Acquisition session, participants will receive an introduction to the CSL Sona-Match program used for visual-acoustic biofeedback. As with ultrasound biofeedback, this introduction will last roughly 20–25 min and will be guided by a standard script that has been uploaded to the Open Science Framework site linked below. The Sona-Match software presents a dynamic display of the speech signal in the form of a real-time LPC (Linear Predictive Coding) spectrum. It also allows the clinician to load a template representing an appropriate pattern of formant heights for a particular sound, which can be superimposed over the dynamic LPC spectrum of the child's speech. Participants will initially be familiarized with the technology by being encouraged to produce a variety of sounds and observe how the formants (“peaks” or “bumps”) move when different sounds are produced. They will then be familiarized with the concept of matching formant templates through an exercise in which the clinician presents a template for a vowel that the child can articulate correctly, then cues the child to try different vowel sounds and guess the target sound based on the closest match. Once a participant demonstrates comprehension of this procedure for matching a template, the target formant configuration for /ɹ/ will be introduced with static images and videos. Participants will be taught that correct /ɹ/ productions are characterized acoustically by a low frequency of the third formant, which creates proximity between the second and third formants. To check comprehension, participants will be asked to differentiate between correct and incorrect /ɹ/ as seen in the visual-acoustic display.

As above, pre-practice and structured practice will have the same basic characteristics as in non-biofeedback treatment, with the exception that the clinician will cue the child to match the visual formant target. It is important to note that articulator placement cues (following the same list of cues as the MBT condition) will be made available during visual-acoustic biofeedback practice, even though the visual display does not provide direct information about articulation. When the clinician cues the child to do something with the tongue (e.g., retract the tongue root), the child and clinician can visually observe the acoustic consequences of that modification and judge whether it brought them closer to an acoustically correct /ɹ/. A formant template will be selected for each child to match during practice. Because formant heights are influenced by vocal tract size, a procedure will be followed to identify the best-matching option from a library of formant templates collected from typically developing children of different ages and heights. Because participants with RSE may not be able to produce any correct /ɹ/ sounds, this matching procedure will be performed based on productions of the vowel /i/, and participants will be assigned a target /ɹ/ template from the typically developing child whose /i/ production best matched their own. As was the case for ultrasound tongue shape templates, if the child starts to achieve perceptually accurate /ɹ/ in treatment, the template based on another child’s production can be replaced with a template representing a freeze-frame of the participant’s own best approximation of /ɹ/.

### Stimuli and scoring

The /ɹ/ sound has somewhat different articulatory and/or acoustic characteristics in different coarticulatory contexts; these contexts may be differentially affected across children with /ɹ/ misarticulation [10]. In the present study, target variants will not be customized on a per-participant basis; instead, all sessions will feature stimuli selected to represent five major phonetic contexts. These contexts are the following: syllabic /ɝ/ as in *bird*, postvocalic /ɹ/ in a front vowel context as in *deer* or *chair*, postvocalic /ɹ/ in a back vowel context as in *star* or *door*, prevocalic /ɹ/ in a front vowel context as in *red* or *ran*, and prevocalic /ɹ/ in a back vowel context as in *rob* or *rude*.

In pre-practice for Phase 0 (Dynamic Assessment) and Phase 1 (Acquisition), stimuli will be drawn from a fixed list consisting of up to three syllables representing each of the five contexts identified above. One syllable will be randomly selected to represent each context. In Phase 2 (Generalization), stimuli for pre-practice will be drawn from the list of words to be targeted in structured practice.

During structured practice, all stimuli will be presented and clinician responses recorded using our custom open-source *Challenge Point Program* (CPP) software [35]. The syllables or words targeted in each session will be randomly selected from a master list by the CPP software. In Phase 1 (Acquisition), the software will select one syllable to represent each of the five variants listed above, resulting in a total of five targets per session. In Phase 2 (Generalization), the software will select two syllables/words from each of the five variants listed above, resulting in a total of ten targets per session. Practice will occur in blocks of ten consecutive trials on the same syllable or word, after which a new syllable or word will be addressed (e.g., ten trials of /ɹa/ followed by ten trials of /ɹu/). If the participant moves up in the adaptive difficulty hierarchy detailed below, new items will be selected, potentially resulting in more than ten words or syllables per session. After the software presents a stimulus and the participant produces it, the clinician will score the response as 0 or 1 based on their clinical impression of an incorrect or correct production of /ɹ/. Only fully correct productions will be scored as 1; distorted productions will be scored as 0.

In Phase 2 (Generalization) only, following each block of ten trials, the software automatically tallies the scores entered by the clinician and uses them to make adaptive changes in task difficulty for the next block. This reflects a goal of keeping learners at a level of difficulty that is neither too hard nor too easy in order to maximize opportunities for learning during speech practice; this “challenge point” concept is drawn from previous motor learning research [36, 37]. If a participant produces eight or more correct responses in a block, the next block will feature an increase in difficulty; five or fewer correct responses will prompt a decrease in difficulty; and six or seven correct responses will trigger no change in difficulty. At the end of a session, the parameter settings are saved, and the next session begins at a level determined by the participant’s performance at the end of the previous session. A total of 15 different levels of increasing difficulty are built into the program.

The parameters used to adjust task difficulty include the linguistic complexity of the string in which the /ɹ/ target is elicited, the frequency with which verbal feedback and/or biofeedback are made available, and the mode of elicitation. These parameters will be adjusted on a rotating basis, such that a participant’s first increase in difficulty will involve an increase in linguistic complexity, the next a reduction in feedback frequency, and the next a change in mode of elicitation. Manipulations of stimulus complexity involve changes in the number of syllables per word, the presence or absence of the competing phonemes /l/ and /w/, and the presence or absence of a carrier phrase or sentence context; at the highest level, participants are asked to formulate their own sentence containing a target word. Manipulations of feedback include a gradual reduction in the frequency with which KP and KR feedback are provided (see details in Verbal Feedback Provided by Clinicians section below); at the highest levels, participants are asked to self-evaluate instead of relying on the clinician’s feedback. In biofeedback treatment conditions, the frequency with which the biofeedback display is made available is decreased in tandem with the reductions in verbal feedback frequency (from 80% at the highest level to 0% at the lowest level). Biofeedback frequency reduction will be achieved by minimizing the Sona-Match software in the visual-acoustic biofeedback condition, or by turning the probe off in the ultrasound biofeedback condition. The final parameter to be adjusted is mode of elicitation. At the lowest level, participants will simply be prompted to read each stimulus word; at higher levels, participants will be asked to produce each word or phrase with a prosodic manipulation such as an interrogative or exclamatory intonation pattern. These prosodic manipulations will initially be applied in a blocked fashion (i.e., all items in a block share the same intonation contour) and then in a randomized fashion.

In addition to the within-session changes described above, the CPP allows for performance-based adjustments to the schedule of stimulus presentation that are applied on a between-session basis. Specifically, if the participant’s cumulative accuracy in structured practice in a given session equals or exceeds 80%, the next session will feature a change from fully blocked practice (i.e., each block elicits 10 trials of a single word, and all words representing a given phonetic context for /ɹ/ are elicited in sequence) to random-blocked practice (i.e., each block elicits 10 trials of a single word, but across blocks, different words and phonetic contexts for /ɹ/ can appear in random order). If the child again achieves at least 80% accuracy at the session level, the schedule will again change to feature fully random practice (i.e., different words and variants are represented within each block of 10 trials).

### Verbal feedback provided by clinicians

During practice, each trial is designated to be followed by no feedback, by verbal feedback on the accuracy of /ɹ/ production (i.e., Knowledge of Results feedback, or KR) or by qualitative feedback on /ɹ/ production (i.e., Knowledge of Performance feedback, or KP). To increase fidelity in treatment delivery, the CPP software will provide prompts indicating when and what type of feedback should be provided during structured practice components of treatment. In Phase 1 (Acquisition), KP feedback will be prompted after every other trial, such that the clinician will provide qualitative feedback appropriate for the participant’s treatment condition. In Phase 2 (Generalization), the CPP will prompt a mix of KP and KR feedback, with the frequency of each feedback type changing over the different levels in the CPP complexity hierarchy. When a trial is designated to receive KR feedback, the CPP will automatically display a feedback message (e.g., “Great job”/“Try again”) based on the score for that trial entered by the treating clinician. The clinician may additionally verbalize this feedback, but this is not required. When a trial is designated to receive KP feedback, the CPP will prompt the clinician to provide qualitative verbal feedback. For the purpose of the present study, KP feedback has been operationalized to include 2–3 specific components. First, the clinician must reference what the child is doing or should be doing with the articulators (e.g., “I like the way you kept the front of the tongue up” or “Remember that the back of your tongue should be pulled back for /ɹ/”). Second, if the participant is in one of the biofeedback treatment conditions, the KP feedback must also make reference to what is seen on the visual display. (To make this possible, the CPP software restricts prompts for KP feedback to occur only on trials in which the biofeedback display is supposed to be made available.) The final component of KP feedback is an explicit verbal model of correct production that the clinician provides as a prompt to encourage correct production in the next trial.

### Clinician training

To become familiar with critical information about the study and the technologies used in it, treating clinicians will initially review a series of informational modules in Powerpoint format generated by the study PIs. A total of ten separate modules cover a range of topics including how /ɹ/ is produced, an overview of ultrasound technology, an overview of visual-acoustic technology, and a guide to using the CPP software described above. Three of the modules provide detailed information about how to cue the /ɹ/ sound in each of the three treatment conditions investigated (MBT, cueing with ultrasound, and cueing with visual-acoustic biofeedback); for these three, the Powerpoint has been augmented into a screencast video with one of the PIs providing voiceover narration. Treating clinicians will meet individually with their local PI after completing the training modules so that any outstanding questions can be discussed and resolved.

To obtain hands-on practice in treatment delivery, new clinicians will be required to conduct at least 2 sessions in each treatment condition under the direct supervision of their local PI, who will provide feedback during and after each session. (Pilot participants or students posing as participants will act as the recipients of treatment for this purpose.) The other clinical site will perform fidelity review of at least one pilot session in each treatment condition to ensure that equivalent methods are being used across sites.

To minimize bias on the part of treating clinicians, all training materials have been reviewed to ensure that no treatment approach is advanced as more effective than other alternatives. The training materials accurately characterize MBT as an evidence-based approach that represents the current standard of care. The PI of each site will act to maintain equipoise in laboratory discussions of MBT and biofeedback treatment methods.

### Treatment fidelity

Treating clinicians’ fidelity in adhering to standard protocols will be evaluated through review of screen-recorded video and audio records from a subset of sessions from each participant in each phase of treatment. To preserve blinding at the central site, the two clinical sites will perform fidelity checks for one another. For Phase 0 (Dynamic Assessment), which consists of a single session, fidelity checks will be completed on 20% of participants per site. For Phase 1 (Acquisition), one of the three sessions will be randomly selected for fidelity checking for each participant. For Phase 2 (Generalization), one session will be randomly selected from the first half and one from the second half of treatment for each participant. Due to the lengthy nature of treatment sessions, fidelity checks will be performed on a 50-trial subset randomly selected from within the full session duration.

In each fidelity check, a trained research assistant will review the video record of the clinician’s behavior and compare it against an output record reflecting trial-by-trial prompts generated by the CPP software. For each trial, the CPP output records the following information: (1) whether biofeedback should have been made available or withheld, if applicable; (2) whether the clinician should have provided a verbal model before the trial; (3) whether KP feedback should have been provided or withheld, and (4) whether the client should have been prompted to self-evaluate their production. In each case, the research assistant will score whether the clinician’s behavior accords with the parameter setting indicated by the software. If KP feedback was provided, the research assistant will additionally score whether the clinician’s verbal feedback included the three components indicated above (reference to the observed or desired articulatory behavior, reference to the visual display for biofeedback trials, and provision of a verbal model).

### Recording and equipment

Each site will use 64-bit Dell PCs operating Windows 10 with relevant software for all treatment conditions and evaluation tasks. Equipment for obtaining audio recordings has been standardized across sites. All recordings will be obtained with a head-mounted microphone (AKG C520 Professional Head-Worn Condenser microphone) positioned so the microphone arm is perpendicular to the corner of the mouth. The audio signal from the head-mounted microphone will be routed through a Behringer UMC 404HD audio interface and then to a Marantz solid-state digital recorder; in the visual-acoustic biofeedback condition, an additional output of the Behringer will be connected via line in to the CSL model 4500B hardware used to generate the visual-acoustic biofeedback display. Gain settings on both the audio interface and the digital recorder are standardized across sites, using a predetermined range within which gain can be adjusted on a by-speaker basis to accommodate individual differences in loudness. As a backup, all study activities will also be captured in screenrecorded video. All recordings will be obtained at a 44,100 Hz sampling rate with 16-bit encoding.

### Outcomes measurement

For our primary outcome measure, we will assess acoustic change in children’s /ɹ/ production in a subset of treatment trials produced over the 3 days of Phase 1 (Acquisition). In each block of ten trials, the ninth trial will be elicited with no feedback (KR, KP, or visual biofeedback). These trials, which will be flagged in the acoustic record by a preceding pure-tone burst, will be manually marked with a textgrid in Praat [38] and then submitted to a forced aligner. The resulting phoneme-level annotation will be verified and adjusted as needed by trained research assistants. An automated script [39] will then be used to extract the second and third formant frequencies (F2 and F3) from a 50 ms window at the center of the interval identified as the phoneme /ɹ/. F3-F2 distance, which is smaller in correct /ɹ/, will serve as the primary acoustic correlate of accuracy. This measure was selected because it provides some degree of correction for differences in vocal tract size and has also been shown to correlate strongly with perceptual ratings of /ɹ/ production accuracy [40, 41]. Research assistants conducting this analysis will be blind to treatment condition.

Our secondary outcome measure will evaluate /ɹ/ production accuracy in a standard probe (50 words containing /ɹ/ in various phonetic contexts) elicited before treatment and again in a post-treatment assessment visit administered within approximately 1 week of the end of Phase 2 (Generalization). To enhance the real-world relevance of this measure, we will use blinded naïve listeners’ ratings of /ɹ/ production accuracy. (Perceptual ratings are not feasible for Phase 1 because within-treatment trials may be prolonged or otherwise unnatural, posing a confound for accuracy ratings provided by untrained listeners.) Following protocols refined in our previous research [42, 43], we will split each word probe recording into 50 word-level productions and pool these productions across speakers and time points. These stimuli will then be presented in a randomized order for online listeners who originate from US-based IP addresses and report speaking American English as their native language. These listeners will be blind to speaker identity and treatment condition, but they will see an orthographic representation of the word being produced in each trial; they will be instructed to rate the accuracy of the /ɹ/ sound in each word in a binary fashion (correct/incorrect) while disregarding other sounds in the word. Per previous research, ratings will be collected until at least 9 unique listeners have rated each token. The proportion of “correct” ratings out of the total number of ratings (p̂-correct), which correlates strongly with both expert listeners’ ratings and acoustic measures [42, 43], will serve as our primary perceptual measure of /ɹ/ production accuracy.

Finally, a survey assessing the social, emotional, and academic impact of RSE will be collected from participants’ caregivers immediately before and after treatment [1]. The survey consists of 11 items (e.g., “My child’s speech sounds different from the speech of other children his or her age”; “My child’s speech has an impact on his or her academic performance”) that are presented in connection with a 5-point Likert scale, where a score of 5 represents strong agreement and a score of 1 represents strong disagreement. Responses will be combined using a Generalized Partial Credit Model [44], in which individual item scores are summed into an overall impact score, with weighting to reflect a stronger or weaker association between each item and the total score.

### Data analysis plan

All analyses will be conducted following the intent-to-treat principle, comparing participants based on the treatment assigned regardless of subsequent exposure. To maintain the full sample, missing data will be multiply imputed for participants who drop out of the study using the *mice* package [45] in the R statistical software language [46]. This approach will capitalize on information collected from these participants prior to their loss to follow-up.

To assess impact on our primary outcome, the acoustic accuracy measure of F3-F2 distance, we will fit a multilevel model using Phase 1 (Acquisition) data. The primary effect of interest is a comparison between MBT and biofeedback treatments, with visual-acoustic and ultrasound biofeedback types pooled for this analysis. This initial model will also adjust for site, performance response category (high responder versus low responder based on Dynamic Assessment), Acquisition day (1, 2, or 3), and pre-treatment accuracy (mean F3-F2 distance across /ɹ/ sounds in the word probe elicited in the first pre-treatment evaluation). If a within-session time trend is supported by the data, we will extend the model to account for time trends across trials within each day of treatment. Random intercepts will be included to reflect the fact that observations are nested within speakers and words, and random slopes that improve overall model fit (for speaker, acquisition day; for word, performance response category and pre-treatment accuracy) will be retained following model comparison using likelihood ratio tests.

Likelihood ratio tests will also be used to test the hypothesis of an advantage for biofeedback over MBT. Tests will be performed in two different model specifications. In one, the overall effect across days will be tested by focusing on the coefficient on the treatment variable. The other specification will interact the treatment variable with time in order to test whether the effect varies across days, since the hypothesized advantage for biofeedback over MBT could emerge as early as the first day or as late as the third day of Phase 1 (Acquisition). Although a positive effect of biofeedback is hypothesized, two-sided hypothesis tests will be used to be conservative.

Our second analysis will fit a linear mixed-effects model using word probe data collected in one immediate pre-treatment and one immediate post-treatment session. The outcome variable will reflect the proportion of naïve listeners who rated the /ɹ/ sound in a given production as correct. The model will include an indicator for time point (pre- versus post-treatment) and for treatment group (biofeedback versus MBT), as well as the interaction between them. As was the case for our primary measure, the model will adjust for site, performance response category based on Dynamic Assessment, and pre-treatment accuracy. Random intercepts for speaker, item (the 50 target words making up the probe measure), and listener will be included. Random slopes of response category and pre-treatment accuracy on word will be examined and included only as warranted by model comparison. As above, likelihood ratio tests will be used to assess the significance of the overall treatment effect. Again, while a positive effect of biofeedback is hypothesized, we will use a two-sided hypothesis test to be conservative.

For this second model, we will additionally test for the presence of a significant time-by-condition interaction in which the magnitude of improvement associated with biofeedback significantly is allowed to vary with time. If we observe a difference in our primary measure (Acquisition) but not our secondary measure (Generalization), this will be interpreted as evidence that biofeedback is more efficient, but not more effective, than MBT.

The final analysis will evaluate changes in impact scores from the 11-item survey assessing the social, emotional, and academic consequences of RSE from pre- to post-treatment time points. A mixed model similar to that used in the previous analyses will be fit including time (pre- versus post-treatment), treatment group (biofeedback versus MBT), and the time-treatment interaction as the primary predictors. The model will also adjust for site, performance response category based on Dynamic Assessment, and pre-treatment accuracy. We will again estimate the interaction between time and treatment group to evaluate whether the functional impact of treatment differs across biofeedback versus MBT conditions.

## Discussion

### Potential significance

When the proposed data collection is complete, we will have measured both short-term acquisition and longer-term generalization changes in /ɹ/ production accuracy in two groups of children randomly assigned to receive MBT or biofeedback treatment. Based on our previous findings, we hypothesize that both groups will improve over the course of treatment, but we also predict significant between-group differences. For our Acquisition measure, we predict that the mixed-effects models will show a significant advantage for biofeedback over MBT as early as the first day of Phase 1, and no later than the final day. For the Generalization measure, we predict a significant time-by-condition interaction in which pre-post gains associated with biofeedback significantly exceed those observed in MBT. To evaluate whether functional impact differs across treatment types, we will look for the same interaction in survey scores. If these hypotheses are supported, it will constitute high-quality evidence that biofeedback outperforms MBT in both efficiency and efficacy. If we observe a difference in Acquisition but not Generalization or functional impact, this will constitute evidence that biofeedback is more efficient, but not more effective, than MBT.

This research has the potential to be clinically significant because it will provide the high-quality data needed to make an evidence-based recommendation regarding the adoption of biofeedback for children with RSE, who occupy disproportionate resources on the SLP caseload. In the United States, 89% of SLPs in the school setting report working with children with speech sound disorder, and the average caseload includes 18 children with this condition [47]. By identifying the most efficient treatment approach, this research could in principle help move children with RSE off the caseload faster, thus improving their social and academic outcomes, reducing treatment costs, and freeing up resources for other children in need of SLP services [8]. If our hypothesis of an advantage for biofeedback is supported, we will follow up with larger studies evaluating the effectiveness of biofeedback treatment in real-world conditions, such as a school setting. Even if we find no significant advantage for biofeedback over MBT, this research still has the potential to have significant clinical impact. Because the proposed trial is well-powered, a null finding would support the clinically important conclusion that the additional expense associated with adopting biofeedback may not be justified. In addition, it is important to keep in mind that most participants with RSE have a history of months or years of non-response to traditional treatment methods [13, 14, 16]. If traditional treatment as operationalized in our study (MBT) is shown to yield the moderate to large effect size we typically see for biofeedback, a publication describing the method would in itself be a meaningful addition to the literature.

### Potential limitations

The present study has a number of limitations. First, for our primary analysis, we plan to pool together ultrasound and visual-acoustic types of biofeedback, which have shown similar evidence of efficacy in previous literature. However, very few studies have directly compared the two types of biofeedback against each other, and it is possible that one type is simply more effective than the other. To address this possibility, we will evaluate the relative effect of visual-acoustic versus ultrasound biofeedback treatment in an exploratory analysis that could inform future studies comparing the two.

Second, we have made every effort to maintain equipoise in our operationalization and execution of treatment, but it is impossible to completely eliminate the possibility that either clinicians or participants/parents could carry their own preference for one treatment over another. The novelty of the technology used in biofeedback may be a source of motivation for participants. This could result in greater gains in the biofeedback condition that are not directly attributable to the visualization component of the treatment, but rather to the participant’s increased engagement in the therapy process. We do not have a definite solution for this issue. However, one approach we are taking is to examine not only whether biofeedback works, but why it works—specifically, by testing hypotheses about the interaction of participants’ profiles of sensory acuity with their response to different types of biofeedback treatment. This is outside of the scope of the present paper but could help address this limitation in our broader program of research.

Third, our primary outcome measure assesses learning on a very short time frame, namely by examining changes within the treatment session during the first phase of intervention. Even our longer-term generalization measure examines performance immediately after the conclusion of treatment; no longer-term follow-up measures are planned. In addition, our analyses focus on /ɹ/ production accuracy at the word rather than the sentence or conversational level. We are collecting sentence-level data and plan to examine these in a supplementary analysis. In general, though, the addition of longer-term follow-up measures and broader assessment of generalization represents an important goal for future research.

Finally, even if we find that our hypothesis of an advantage for biofeedback is supported, we still face a challenge in translating this finding into clinical practice due to barriers to adoption of biofeedback—notably the high cost of the equipment and the need for specialized training to use it correctly. Costs of equipment are declining as technology advances, but it remains a significant obstacle for the average clinician. One avenue we are pursuing to address this limitation is a separate program of research developing a low-cost app to deliver visual-acoustic biofeedback treatment [48]. In addition, throughout this and other studies, we are working to develop training materials that can be shared with clinicians interested in adopting biofeedback, which could make access to training less of an obstacle.

## Data Availability

Materials referenced in this protocol are available in an Open Science Framework repository at https://osf.io/6qs4d/, DOI 10.17605/OSF.IO/6QS4D. After collection, de-identified participant-level data will be made available in the same location.

## Abbreviations

CAS: Childhood Apraxia of Speech
CELF-5: Clinical Evaluation of Language Fundamentals - Fifth Edition
CPP: Challenge Point Program (software)
CSL: Computerized Speech Lab (hardware/software)
GFTA-3: Goldman Fristoe Test of Articulation - Third Edition
KP: Knowledge of Performance (feedback)
KR: Knowledge of Results (feedback)
LPC: Linear Predictive Coding (spectrum)
MBT: Motor-Based Treatment
MR: Magnetic Resonance (imaging)
RSE: Residual Speech Errors
SLP: Speech-Language Pathologist
WASI-2: Wechsler Abbreviated Scales of Intelligence – Second Edition

## Footnotes

1 Across all treatment conditions, the first treatment session in Phase 1 (Acquisition) will feature roughly 25 min of additional condition-specific training. For the MBT condition, this will consist of revisiting the content introduced during Dynamic Assessment (tongue shapes for /ɹ/, etc.) in greater detail.

## References

[1] Hitchcock E, Harel D, McAllister BT (2015). Social, emotional, and academic impact of residual speech errors in school-aged children: a survey study. Semin Speech Lang.

[2] Crowe Hall BJ (1991). Attitudes of fourth and sixth graders towards peers with mild articulation disorders. Lang Speech Hear Serv Sch.

[3] McCormack J, McLeod S, McAllister L, Harrison LJ (2009). A systematic review of the association between childhood speech impairment and participation across the lifespan. Int J Speech Lang Pathol..

[4] Felsenfeld S, Broen PA, McGue M (1992). A 28-year follow-up of adults with a history of moderate phonological disorder: linguistic and personality results. J Speech Lang Hear Res.

[5] Shriberg LD, Paul R, Flipsen P (2009). Childhood speech sound disorders: from postbehaviorism to the postgenomic era. Speech sound disorders in children.

[6] Culton GL (1986). Speech disorders among college freshmen: a 13-year survey. J Speech Hear Disord.

[7] Flipsen P (2015). Emergence and prevalence of persistent and residual speech errors. Semin Speech Lang.

[8] Ruscello DM (1995). Visual feedback in treatment of residual phonological disorders. J Commun Disord.

[9] Volin RA (1998). A relationship between stimulability and the efficacy of visual biofeedback in the training of a respiratory control task. Am J Speech Lang Pathol..

[10] McAllister Byun T, Hitchcock ER (2012). Investigating the use of traditional and spectral biofeedback approaches to intervention for /r/ misarticulation. Am J Speech Lang Pathol..

[11] McAllister Byun T, Campbell H (2016). Differential effects of visual-acoustic biofeedback intervention for residual speech errors. Front Hum Neurosci.

[12] McAllister BT (2017). Efficacy of visual-acoustic biofeedback intervention for residual rhotic errors: a single-subject randomization study. J Speech Lang Hear Res.

[13] Preston JL, McCabe P, Rivera-Campos A, Whittle JL, Landry E, Maas E (2014). Ultrasound visual feedback treatment and practice variability for residual speech sound errors. J Speech Lang Hear Res.

[14] Preston JL, Leece MC, Maas E (2017). Motor-based treatment with and without ultrasound feedback for residual speech sound errors. Int J Lang Commun Disord.

[15] Preston JL, McAllister T, Phillips E, Boyce S, Tiede M, Kim JS (2019). Remediating residual rhotic errors with traditional and ultrasound-enhanced treatment: a single-case experimental study. Am J Speech Lang Pathol.

[16] McAllister Byun T, Hitchcock ER, Swartz MT (2014). Retroflex versus bunched in treatment for rhotic misarticulation: evidence from ultrasound biofeedback intervention. J Speech Lang Hear Res.

[17] Sugden E, Lloyd S, Lam J, Cleland J (2019). Systematic review of ultrasound visual biofeedback in intervention for speech sound disorders. Int J Lang Commun Disord.

[18] Hodges NJ, Franks IM (2001). Learning a coordination skill: interactive effects of instruction and feedback. Res Q Exerc Sport.

[19] Maas E, Robin DA, Austermann Hula SN, Freedman SE, Wulf G, Ballard KJ (2008). Principles of motor learning in treatment of motor speech disorders. Am J Speech Lang Pathol..

[20] Van Riper C. Speech Correction: Principles and Methods, vol. 1. Englewood Cliffs: Prentice-Hall; 1939.

[21] Van Riper C. Speech Correction: Principles and Methods, vol. 9. Englewood Cliffs: Prentice-Hall; 1996.

[22] Faul F, Erdfelder E, Lang A-G, Buchner A (2007). G*power 3: a flexible statistical power analysis program for the social, behavioral, and biomedical sciences. Behav Res Methods.

[23] Harris PA, Taylor R, Thielke R, Payne J, Gonzalez N, Conde JG (2009). Research electronic data capture (REDCap)--a metadata-driven methodology and workflow process for providing translational research informatics support. J Biomed Inform.

[24] Harris PA, Taylor R, Minor BL, Elliott V, Fernandez M, O’Neal L (2019). The REDCap consortium: Building an international community of software platform partners. J Biomed Inform.

[25] Wechsler D (2011). Wechsler abbreviated scales of intelligence.

[26] Wiig E, Semel E, Secord W (2013). Clinical evaluation of language fundamentals—fifth edition (CELF-5).

[27] Goldman R, Fristoe M (2015). Goldman-Fristoe test of articulation.

[28] Shriberg LD, Lohmeier HL, Campbell TF, Dollaghan CA, Green JR, Moore CA (2009). A nonword repetition task for speakers with misarticulations: the syllable repetition task (SRT). J Speech Lang Hear Res JSLHR.

[29] Bowers L, Huisingh R (2011). Linguisystems articulation test.

[30] Thoonen G, Maassen B, Gabreels F, Schreuder R, de Swart B (1997). Towards a standardised assessment procedure for developmental apraxia of speech. Eur J Disord Commun.

[31] Thoonen G, Maassen B, Gabreels F, Schreuder R (1999). Validity of maximum performance tasks to diagnose motor speech disorders in children. Clin Linguist Phon.

[32] Miccio AW (2002). Clinical problem solving: assessment of phonological disorders. Am J Speech Lang Pathol.

[33] Boyce SE (2015). The articulatory phonetics of /r/ for residual speech errors. Semin Speech Lang.

[34] Klein HB, McAllister Byun T, Davidson L, Grigos MI (2013). A multidimensional investigation of children’s /r/ productions: perceptual, ultrasound, and acoustic measures. Am J Speech Lang Pathol..

[35] McAllister Byun T, Hitchcock E, Ortiz J (2014). Challenge-R.

[36] Guadagnoli MA, Lee TD (2004). Challenge point: a framework for conceptualizing the effects of various practice conditions in motor learning. J Mot Behav.

[37] Rvachew S, Brosseau-Lapré F (2012). Developmental phonological disorders: foundations of clinical practice.

[38] Boersma P, Weenink D (2019). Praat: doing phonetics by computer. 6.1.06.

[39] Lennes M (2003). Collect formant data from files [internet].

[40] Campbell Heather, Harel Daphna, Hitchcock Elaine, McAllister Byun Tara (2017). Selecting an acoustic correlate for automated measurement of American English rhotic production in children. International Journal of Speech-Language Pathology.

[41] Dugan SH, Silbert N, McAllister T, Preston JL, Sotto C, Boyce SE (2019). Modelling category goodness judgments in children with residual sound errors. Clin Linguist Phon.

[42] McAllister Byun T, Halpin PF, Szeredi D (2015). Online crowdsourcing for efficient rating of speech: a validation study. J Commun Disord.

[43] McAllister Byun T, Harel D, Halpin PF, Szeredi D (2016). Deriving gradient measures of child speech from crowdsourced ratings. J Commun Disord.

[44] Muraki Eiji (1997). A Generalized Partial Credit Model. Handbook of Modern Item Response Theory.

[45] van Buuren S, Groothuis-Oudshoorn K (2011). Mice: multivariate imputation by chained equations in R. J statistical software.

[46] R Core Team (2017). R: a language and environment for statistical computing.

[47] ASHA (2016). ASHA 2016 Schools survey: SLP caseload characteristics report [internet].

[48] McAllister Byun T, Campbell H, Carey H, Liang W, Park TH, Svirsky M (2017). Enhancing intervention for residual rhotic errors via app-delivered biofeedback: a case study. J Speech Lang Hear Res.

